# Who and where are the uncounted children? Inequalities in birth certificate coverage among children under five years in 94 countries using nationally representative household surveys

**DOI:** 10.1186/s12939-017-0635-6

**Published:** 2017-08-18

**Authors:** Amiya Bhatia, Leonardo Zanini Ferreira, Aluísio J. D. Barros, Cesar Gomes Victora

**Affiliations:** 1 Harvard T.H. Chan School of Public Health, 677 Huntington Ave, Boston, MA 02115 USA; 2 International Center for Equity in Health, Federal University of Pelotas, Mal. Deodoro, 1160, 3d Floor, Pelotas, RS 96020-220 Brazil

**Keywords:** Birth certificates, Vital statistics, Health equity, Child health surveys, Global health, Socioeconomic factors

## Abstract

**Background:**

Birth registration, and the possession of a birth certificate as proof of registration, has long been recognized as a fundamental human right. Data from a functioning civil registration and vital statistics (CRVS) system allows governments to benefit from accurate and universal data on birth and death rates. However, access to birth certificates remains challenging and unequal in many low and middle-income countries. This paper examines wealth, urban/rural and gender inequalities in birth certificate coverage.

**Methods:**

We analyzed nationally representative household surveys from 94 countries between 2000 and 2014 using Demographic Health Surveys and Multiple Indicator Cluster Surveys. Birth certificate coverage among children under five was examined at the national and regional level. Absolute measures of inequality were used to measure inequalities in birth certificate coverage by wealth quintile, urban/rural residence and sex of the child.

**Results:**

Over four million children were included in the analysis. Birth certificate coverage was over 90% in 29 countries and below 50% in 36 countries, indicating that more than half the children under five surveyed in these countries did not have a birth certificate. Eastern & Southern Africa had the lowest average birth certificate coverage (26.9%) with important variability among countries. Significant wealth inequalities in birth certificate coverage were observed in 74 countries and in most UNICEF regions, and urban/rural inequalities were present in 60 countries. Differences in birth certificate coverage between girls and boys tended to be small.

**Conclusions:**

We show that wealth and urban/rural inequalities in birth certificate coverage persist in most low and middle income countries, including countries where national birth certificate coverage is between 60 and 80%. Weak CRVS systems, particularly in South Asia and Africa lead rural and poor children to be systematically excluded from the benefits tied to a birth certificate, and prevent these children from being counted in national health data. Greater funding and attention is needed to strengthen CRVS systems and equity analyses should inform such efforts, especially as data needs for the Sustainable Development Goals expand. Monitoring disaggregated data on birth certificate coverage is essential to reducing inequalities in who is counted and registered. Strengthening CRVS systems can enable a child’s right to identity, improve health data and promote equity.

**Electronic supplementary material:**

The online version of this article (doi:10.1186/s12939-017-0635-6) contains supplementary material, which is available to authorized users.

## Background

A birth certificate is proof of identity, age, and family relationships, and confirms that a child’s birth has been registered. Provided to a child by a civil registry, it allows an individual to make claims of nationality, benefit from government schemes, open a bank account, travel, and vote. Although a birth certificate does not guarantee protection, it can help protect children from abuse and exploitation [1, 2], reduce child marriage, allow inheritance to be claimed, and prevent statelessness [3]. The right to birth registration is affirmed in the United Nations Convention on the Rights of the Child [4] which has been ratified by 195 countries [5]. However, UNICEF estimates suggest nearly 230 million children under age five are not registered at birth and do not have access to a birth certificate [6]. In addition to being denied the rights, social services, and child protection associated with registration, unregistered children are not counted or captured in Civil Registration and Vital Statistics (CRVS) systems which allow an understanding of who is being born and dying [6, 7] and serve to drive policy and planning [8]. CRVS systems also permit the production, and monitoring, of statistics on population health, and enable accountability.

Birth registration is included in the Sustainable Development Goals (SDGs), which have an explicit aim to ensure access to widely accepted, robust identity credentials, and improve vital registration systems [9, 10]. In addition, improved birth registration will also enable the monitoring of other child health goals, including under-five mortality. Although there has been an overall increase in global birth registration rates of children under five from 58 to 65% in the last decade, over 100 countries still do not have functioning systems that can support the registration of births and other life events [3].

In 2013, a UNICEF report showed that socioeconomic status, religion, maternal education, and access to a health facility can determine which children benefit from identification documents [11]. However, there is a paucity of systematic analyses of inequalities in birth certificate coverage in low and middle income countries using nationally representative survey data, and a dearth of efforts to monitor inequalities in birth registration coverage. In this paper, we aim to address this gap in the literature and examine who and where the uncounted children are, thus informing efforts to monitor and strengthen CRVS systems, and improve birth certificate coverage.

## Methods

### Data sources

We analyzed birth certificate and birth registration coverage through nationally representative household surveys, including 48 Demographic Health Surveys (DHS) [12] and 46 Multiple Indicator Cluster Surveys (MICS) [13]. Both DHS and MICS questionnaires asked the caregiver whether each child in the household had a birth certificate, and, in the absence of a certificate, whether each child’s birth had been registered.

We included all surveys with data on birth registration after the year 2005 (including surveys from the third, fourth and fifth wave of MICS and from the fifth and sixth wave of DHS). We selected the most recent survey for each country. All surveys were publicly available and ethical clearance was the responsibility of the institutions that administered the surveys. Countries were categorized by region based on UNICEF regions [14], and by income group at the time of the survey using historical data on World Bank income groups [15].

### Outcomes

Outcomes were constructed using one standard question from DHS and three standard questions from MICS. All outcomes were based on a caregiver report of whether a child had been registered and whether a birth certificate was issued. Although there were small differences between the DHS and MICS questions, both surveys allowed each child to be classified into three groups: “birth registered, with a certificate (which the caregiver is in possession of)”, “birth registered, no certificate”, or “birth not registered”. Details of the questionnaires are available in Additional file 1.

We calculated birth certificate coverage as the primary outcome, defined as the percentage of children under age five (0 to 59 months) with a birth certificate at the time of the survey. The denominator was the population of children under-five surveyed. Since survey sampling was nationally representative, our estimates represent national coverage in each country. Two secondary outcomes were also used in select analyses: first, the proportion of children under five who were reported to have been registered but did not receive a certificate; and second, birth registration coverage, which was calculated as the percentage of children under age five whose birth was reported as registered – this included children with and without a birth certificate. Children under five who were not included in these categories were classified as unregistered.

National estimates were compared to published MICS and DHS reports. All differences between calculated and published estimates were smaller than one percentage point, with the exception - as expected - of the nine MICS 3 surveys included in the analysis due to the standardization of skip patterns between MICS 3 and MICS 4/5.

### Stratification variables

We disaggregated the outcomes in each survey by sex of the child, wealth quintile and urban or rural residence. The wealth index is calculated through principal component analyses of household assets and building characteristics in each country; the first quintile represents the poorest 20% of all households, and the fifth quintile represents the wealthiest 20% of households in the sample in each country [16–18]. The wealth index is comparable across countries in relative terms, e.g. the poorest 20% in each country. However, because the average level of wealth varies by country, the poorest 20% in a given country may well be richer or poorer than the poorest 20% in another country. Urban and rural location was defined by each country before conducting the survey. Additional file 2 shows how these variables were included in the analysis. Sex (male/female) and residence (urban/rural) were included as binary variables, and wealth quintile was included as an ordinal variable.

### Analysis

We calculated point estimates, standard errors and confidence intervals for each outcome, in each country.

Unweighted averages of country estimates were calculated for each UNICEF region and for each World Bank income group at the time of the survey. High income countries (*n* = 3) and countries which were not assigned to an income group (*n* = 2), were excluded from income group estimates.

To examine within country inequalities according to sex of the child and urban/rural residence, we used difference measures. Point estimates, standard errors and confidence intervals were calculated for each difference measure, and confidence intervals were used to assess significance. A negative value indicates lower registration coverage among girls compared to boys, or among children in rural areas compared to urban areas.

To estimate within country wealth inequality, we used the Slope Index of Inequality (SII). Using logistic regression, the SII represents the absolute difference in the fitted value of birth certificate coverage between the highest and the lowest extremes of the wealth distribution [16, 19]. The SII considers all quintiles rather than only the extreme groups. A positive value indicates greater coverage among children in households in the richest wealth quintile compared to the poorest quintile. A small SII indicates a smaller difference in birth certificate coverage between the poorest and richest quintiles. The measures of inequality calculated, their interpretation, and the reference groups for each variable are available in Additional file 2.

All country-level analyses accounted for the multi-stage survey design, including sampling weights and clustering. Analyses were carried out in Stata†/MP13 (StataCorp LP, College Station, Texas, United States).

## Results

Data on birth certificate coverage in 94 countries from seven UNICEF regions were included in the analysis. This included a total of 4,195,238 children. Nineteen surveys were conducted before 2010, and 75 surveys were conducted between 2010 and 2014. Data on World Bank income group at the time of the survey were available for 92 countries (South Sudan and the State of Palestine were not classified into an income group at the time of the survey) of which 32 were low-income countries, 40 were lower-middle income countries, and 17 were upper-middle income countries. The 3 high-income countries were removed from the income group analyses. Disaggregated coverage estimates by sex were available for all 94 countries, and by urban/rural residence and wealth for 92 countries.

**Fig. 1:**
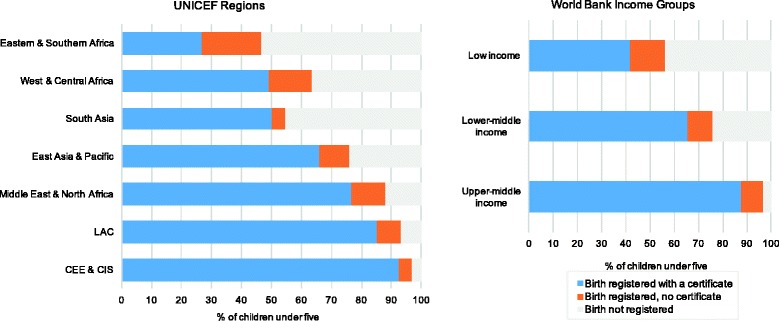
Mean national values of birth certificate and registration coverage among children under five by UNICEF region and World Bank income group. Notes: - Data presented is unweighted. - Estimates for UNICEF regions includes a total of 94 countries; 15 countries from CEE & CIS; 8 countries from. East Asia & Pacific; 17 countries from Eastern & Southern Africa; 15 countries from Latin America and the Caribbean; 10 countries from Middle East & North Africa; 7 countries from South Asia; 22 countries from West & Central Africa. - Estimates for World Bank income group are based on income group at the time of the survey and include a total of 89 countries; 32 low-income countries, 40 lower-middle income countries and 17 upper-middle income countries. Five countries were not included in income group estimates: these included three high income countries (Barbados, Trinidad and Tobago and Uruguay) and two countries which did not have an income group classification at the time of the survey (South Sudan and State of Palestine)

Birth certificate and registration coverage varied by UNICEF region and World Bank income group (Fig. 1). Eastern & Southern Africa had the lowest average birth certificate coverage (26.9%), indicating that almost one in four children under five did not have a birth certificate. Average total registration coverage in this region – which includes children with or without a birth certificate who are reported as registered – was 46.7%, indicating that over half the children surveyed were not registered. In West and Central Africa and South Asia, average birth certificate coverage was just over 50%, however registration coverage was higher in West and Central Africa (63.4%) than South Asia (54.7%). In contrast, birth certificate coverage was highest in Central and Eastern Europe (92.5%) and in Latin America and the Caribbean (85.1%). On average, low-income countries had the lowest birth certificate coverage (41.7%), and the largest proportion of unregistered children while birth certificate coverage was almost double in upper-middle income countries (87.7%).

**Fig. 2:**
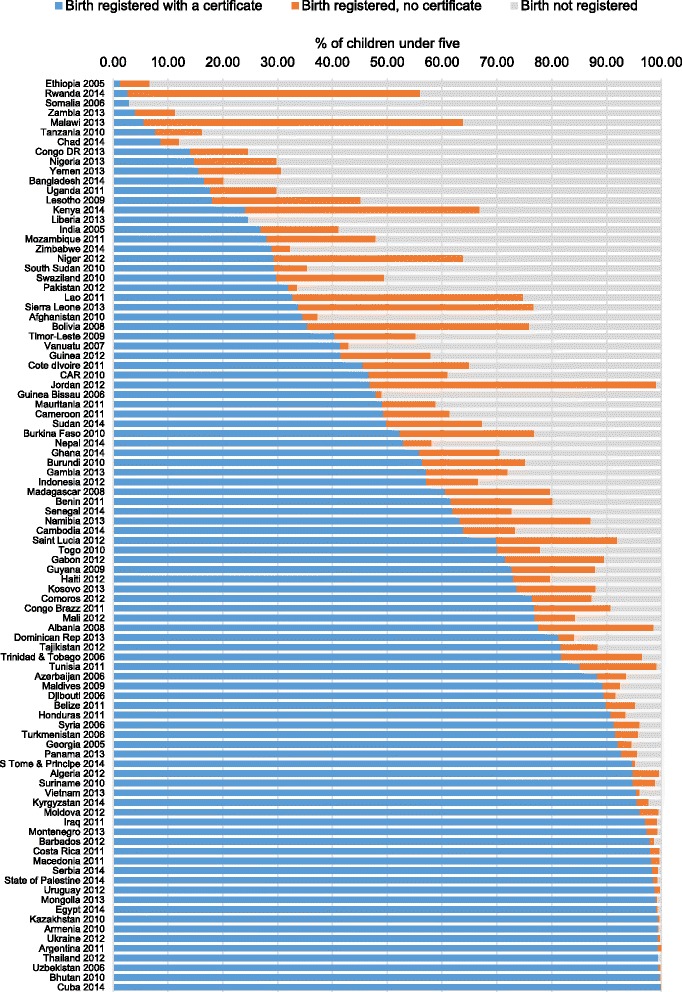
Country level birth certificate and birth registration coverage among children under five in 94 countries

Birth certificate coverage was over 90% in 29 countries - of which coverage was over 98% in 16 countries - and below 50% in 36 countries (Fig. 2). Birth certificate coverage varied widely, ranging from 1.3% in Ethiopia to 99.9% in Cuba. Such variation was also observed within regions and income groups; for example, the region with the lowest coverage - Eastern and Southern Africa – included Comoros with 76.4% coverage, and Ethiopia with the lowest coverage. In Latin America and the Caribbean, coverage was highest in Cuba, yet only 35.4% in Bolivia. In low-income countries, coverage ranged from less than 2% in Ethiopia to 99.5% in Uzbekistan. Similarly, in lower-middle income countries, it ranged from 4.1% in Zambia to 99.7% in Bhutan. The range in birth certificate coverage was smaller for upper-middle income countries, from 63.2% in Namibia to 99.9% in Cuba.

Similarly, total registration coverage ranged from 3% in Somalia to 100% in Cuba and Argentina, and varied widely within regions. In 81 countries children were more likely to be registered with a birth certificate than without. However, in 13 countries more children were registered without receiving a birth certificate than were registered with a certificate. For example, although registration coverage in in Malawi was 63.9%, only 5.6% of children under five had a birth certificate, while 58.3% of children under five were registered without a birth certificate. In Jordan, 99.1% of children under five were registered, however only 46.9% of children under 5 had a birth certificate. Countries with the highest birth certificate coverage had minor differences between the proportion of children registered with or without a birth certificate. Additional file 3 displays birth certificate and registration coverage for each country, as well as the sample size, data source and survey year for each country.

### Wealth quintiles

**Fig. 3:**
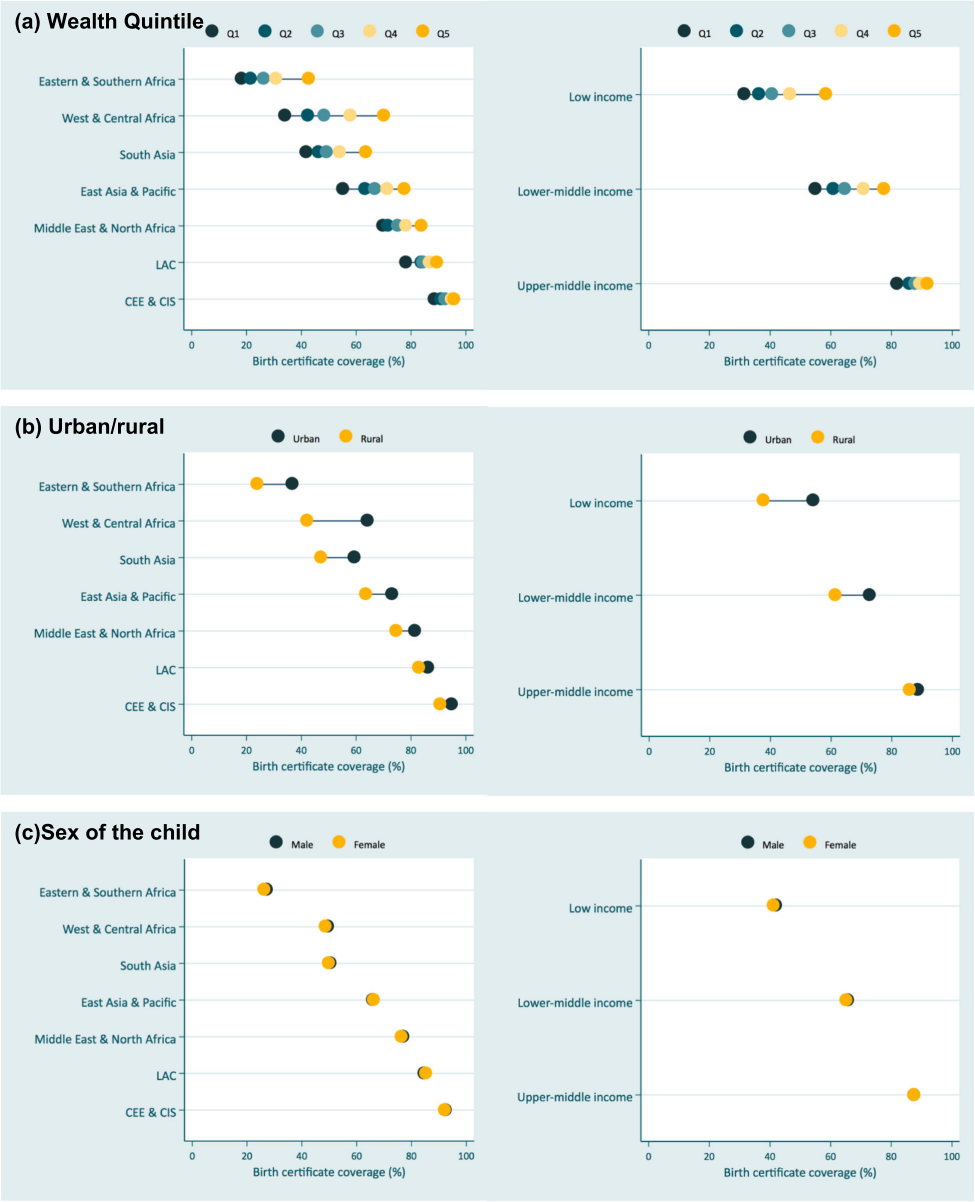
Average inequalities in birth certificate coverage by (**a**) wealth, (**b**) urban/rural residence, and (**c**) gender among children under five by UNICEF region and World Bank income group. Notes: Regional estimates presented are unweighted

Figure 3 shows the average birth certificate coverage by wealth quintile, urban/rural residence and sex of the child for each region and income group. Coverage is systematically lower among children living in households in the poorest, compared to the highest wealth quintile. In spite of high overall coverage some wealth inequality persists in Central and Eastern Europe, Latin America and the Caribbean, and among upper-middle income countries.

For children in households in the poorest wealth quintile, birth certificate coverage was lowest (18.3%) in Eastern & Southern Africa. However, West & Central Africa had the largest gap in coverage, which was 35.7 percentage points lower among children in the poorest quintile compared to children in wealthiest quintile. A similar disadvantage for poor children was prevalent in Eastern & Southern Africa (24.4 percentage points), South Asia (21.7 percentage points), and East Asia & Pacific (22.3 percentage points). Although gaps in coverage by wealth were smaller in Latin America and the Caribbean (11 percentage points) and Central and Eastern Europe (7.2 percentage points), wealth inequalities persisted.

Additional file 4 shows the SlI by wealth for each country. There were significant wealth inequalities in 74 countries where the SII was greater than zero and the confidence interval for the SII did not include the value of zero, indicating a ‘pro-rich’ bias – a lower birth certificate coverage for children in households in the lowest wealth quintile compared to the highest. In 13 countries birth certificate coverage was at least 50 percentage points lower among children in the poorest quintile compared to children in wealthiest quintile, and in 38 countries this difference was at least 20 percentage points. The largest coverage gap was in Pakistan: 72.3 percentage points. In 18 countries there were either no wealth inequalities in birth certificate coverage, or coverage was greater among children in the poorest quintile (e.g. Afghanistan, Costa Rica, Laos, Malawi).

**Fig. 4:**
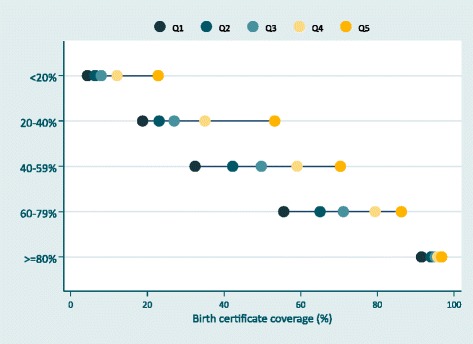
Wealth inequalities by categories of birth certificate coverage. Notes: - 92 countries had data on wealth inequalities. - Birth certificate coverage less than 20% (n=13 countries); 20-40% (n=13 countries); 40-59% (n=16 countries); 60-79% (n=15 countries); >=80% (n=35 countries)

Figure 4 groups countries into categories based on national birth certificate coverage, and examines wealth inequalities for each range of coverage. On average, wealth inequalities are largest when national birth certificate coverage is between 40 and 59%: among these countries, coverage is 37.9 percentage points lower among children in the poorest quintile compared to children in wealthiest quintile. However, large gaps between the rich and the poor are also seen when national birth certificate coverage is 20-40 and 60-79%. When average national coverage is above 80%, the gap between the poorest and richest children shrinks to 5.3 percentage points.

### Urban and rural residence

Our analyses revealed large and consistent disadvantages to rural compared to urban children. Regional estimates showed the largest average gap was in West and Central Africa: 22.1 percentage points. Differences were smaller in other regions: 2 percentage points in Latin America and the Caribbean and 4 percentage points in Central and Eastern Europe. The gap was largest in low-income (16.5 percentage points) and smallest in upper-middle income countries (2 percentage points).

Country level estimates (Additional file 5) suggested that significant inequalities in birth certificate coverage for rural children existed in 60 countries. The widest gap was in Cote d’Ivoire where 73.5% of urban children had a certificate compared to 29.2% of rural children, an absolute difference of 44.3 percentage points. Although urban/rural inequalities decreased as coverage increased past 90%, countries with birth certificate coverage of 70% still had large urban and rural inequalities. For example, national birth certificate coverage was 70.1% in Togo, however rural coverage was 29.6 percentage points lower than in urban areas. Jordan and Thailand were the only countries where birth certificate coverage was statistically significantly higher among children in rural areas, and this difference was largest in Jordan (12.4 percentage points).

### Sex of the child

When average regional values were examined birth certificate coverage was similar for boys and girls (Fig. 3). Similarly, most countries did not show significant differences in coverage by sex of the child (Additional file 6). Coverage was significantly lower among girls compared to boys in eight countries (Sudan, Niger, Namibia, Guinea Bissau, Tajikistan, Costa Rica, Armenia and Thailand), and the coverage gap was largest in Sudan where birth certificate coverage was 3.4 percentage points lower among girls compared to boys. Coverage was significantly higher among girls in three countries (Kyrgyzstan, Sierra Leone and Vanuatu); the largest difference was in Vanuatu where coverage was 6.7 percentage points higher among girls.

## Discussion

We show that a large percentage of children in low and middle-income countries do not have birth certificates, with rural and poor children facing a systematic disadvantage in access to birth certificates in most of the 94 countries included in the analysis. We also show that, at the national level, differences in birth certificate coverage based on the sex of the child were not observed.

The benefits of birth registration are well described in the literature [8, 20, 21], including the role played by registration in poverty reduction by enabling access to cash transfers and social welfare [22, 23]. There is also some research which suggests that improved CRVS performance coincides with improved health [24, 25]. The experience of countries with universal birth certificate coverage demonstrate its benefits for the real time surveillance of births, which - when combined with high quality data on deaths - allows health statistics to be generated and monitored [26]. Our findings show that in most low and middle income countries included in the analysis the realization of both the right to identity for children, and of high quality national data for epidemiologic surveillance, are far from complete. Our findings are consistent with data quality assessments of CRVS systems which suggest that global progress has been slow in the past 30 years, and that most countries with low data quality were in the African or Asian regions [27]. Our findings are also consistent with UNICEF research on birth registration [11], and national and sub-national studies conducted in Nigeria and Ghana which suggest that birth registration is a privilege afforded to a subset of children based on maternal education, income, residential location, and access to primary care [28, 29].

Ethiopia, Rwanda and Somalia had the lowest birth certificate coverage. In Ethiopia, the CRVS system was launched in 2016 which explains the low birth certificate coverage observed in 2005 [30]. In Rwanda, while birth certificate coverage is less than 3 %, birth registration coverage is 56%, and a recent government report highlighted how these records are not being fully utilized by the government for its administrative or social services delivery processes [31]. Reports suggest a functional CRVS system does not exist in Somalia [32]. There is some prior research that explores the reasons behind low registration coverage, which include weak government commitment and poorly funded CRVS systems, which are not comprehensive and are often unable to register births in the hospital/health facility, especially in rural areas [3, 33]. Although registration is free in many countries, the economic cost of registration could include obtaining parental identity documents, official fees, fines for late registration, transportation expenses, and bribes [3]. A registration process which discriminates based on ethnicity, religion, refugee status or single parent status can also affect coverage [3]. Existing studies also suggest that a lack of awareness about registration and its benefits could contribute to varying coverage levels [11]; for example, birth registration in a country can be lower than immunization coverage as caregivers may not see the benefits of registration and child health services may not connected. UNICEF argues that an identity registration system is necessary for social and economic development and is affordable, including for low-income countries [3, 11]. Our findings also show that high coverage is possible in every region and income group.

The results we present begin to answer the question posed in the title of this paper – namely who and where are the uncounted children? We show that the children who are counted and included in a population are not random. Instead, the patterning of who and where the uncounted children are is based on wealth and residential location -- the 74 countries with wealth inequalities in birth certificate coverage and 60 countries with urban/rural inequalities show income, and residential location determine both which children benefit from a birth certificate, and which children are counted in CRVS systems and contribute to the measured birth rate, infant mortality rate and population count.

To understand the unequal distribution of birth certificates - and the unequal access to the right to identity connected to them - Victora and co-authors [34] propose the “inverse-equity” hypothesis. They argue public-health interventions and programs reach those of higher socioeconomic status first, and later affect the poor; they contend that inequities only improve once the rich have achieved access, after which the poor gain greater access. The World Health Organization has also described the bias to serve the better-off first [35]. Our findings show children in the poorest wealth quintile are most likely to be unregistered and - given the focus of social transfers and government schemes on the poor - are also most likely to be eligible for the very services, social protection and government schemes which a lack of registration can prevent them from accessing. Results also suggest that on average, wealth inequalities in birth certificate coverage do not shrink until countries have achieved very high coverage. Notably, even after coverage surpasses 80%, there remains, on average, a 10 percentage point gap between the poorest and wealthiest children. This underscores the importance of policies and programs aimed at raising coverage while preventing an increase in inequality, in the effort to achieve universal birth certificate coverage.

Between-country comparisons of birth certificate coverage are useful to track global progress on birth registration. For example, although Gambia and Benin have similar total birth certificate coverage, the absolute difference in coverage by wealth is 43.4 percentage points in Benin and 5.8 percentage points in Gambia. Such examples show that wealth inequalities in registration are not inevitable, and raise questions about how policies that improve a country’s average for a given health indicator without addressing inequality may neither be fair or nor equitable [35].

Efforts to strengthen registration in Brazil, Bangladesh, Nepal and Ghana have shown success and several countries have implemented efforts to link birth registration to health and education services [8, 25, 36], use technology and mobile phones to assist with registration, and have enacted strong legislative support for CRVS systems [8, 22]. However, for these efforts to address inequalities in coverage instead of solely improving average coverage, within country comparisons of birth certificate coverage among sub-groups of wealth, gender, and urban/rural location are needed to monitor inequality and inform efforts to improve access to birth certificates. For example, national birth certificate coverage is 76.8% in Congo Brazzaville, however there is a 22.9 percentage point difference in coverage between urban and rural children, indicating the need to focus on improving access for rural populations. Disaggregating birth certificate coverage data in Tanzania presents a very different picture; national coverage is only 7.7%, being low among all sub-groups and indicating the need for a widespread national intervention to improve access to birth certificates, including efforts to prevent increasing inequality in access.

Some have described unequal registration as the ‘denominator problem’ [36]. We use nationally representative survey data to estimate national birth certificate coverage, which is typically measured through a country’s CRVS system. In the absence of high quality and functioning CRVS systems, such survey data allow both birth registration coverage as well as who is uncounted to be estimated in a way that many registries are currently unable to. Our findings raise important questions about the magnitude of bias in these systems. Krieger [37] examines the definition of a population in the context of research studies to suggest that the ‘restriction of studies to “easy-to-reach” populations can, owing to selection bias, produce biased estimates of risk, lead to invalid causal inferences, and hamper the discovery of needed etiologic and policy-relevant knowledge’. Given the inequalities our findings show, these arguments are relevant for analyses based on data from CRVS systems. Unlike other sources of vital statistics, such as censuses and household surveys, the administrative data provided from CRVS systems permit the production of statistics on population dynamics, health, and inequalities in service delivery on a continuous basis for the country, and for local administrative subdivisions. The weaknesses of these systems have fostered an over reliance on survey data - as we rely on here - to understand counts of births and deaths, arguably detracting from investments in building universal CRVS systems. Boerma and Stansfield [38] underscore the need to address the underlying causes in the deficiencies in health statistics instead of relying solely on national surveys, and although they emphasize the importance of multiple data sources, they suggest the best data source for mortality is through the CRVS system. AbouZahr and co-authors echo this, and suggest that countries and development partners should reject a dependence on suboptimal data sources to monitor mortality and fertility, and investment in CRVS systems will provide a strong evidence base for health and development policies. This will allow household surveys and censuses to focus on the collection of social, behavioral and disease-specific information [21].

Globally, birth registration did not feature prominently in the Millennium Development Goals, however legal identity and birth registration are included in Sustainable Development Goal 16.9 which states “by 2030, provide legal identity for all, including birth registration” [10]. Many have argued that the identity target in the SDGs is foundational to many of the other goals and indicators, especially goal 17.18 which aims to ‘increase significantly the availability of high-quality, timely and reliable data disaggregated by income, gender, age, race, ethnicity, migratory status, disability, geographic location and other characteristics relevant in national contexts’ and underscores the importance of high quality national data [10]. Given that a birth certificate remains essential proof of registration for the child, inequalities in birth certificate coverage should continue to be monitored to track progress on SDG 16.9, and as efforts to improve national data continue to expand.

This study has several limitations. Selection of the most recent survey may not convey the current picture, however, most of the surveys were conducted after 2010 and selecting the most recent survey does provides the most current survey-based estimate of birth registration available. To address the reliance on survey data for this analysis, CRVS systems - which are currently unable to count vital events - need to be strengthened. Possession of a birth certificate is a self-reported measure and was not verified by the interviewer. The term ‘registered with the civil authorities’ which was part of the questionnaire could have multiple definitions, causing some measurement error, which could be addressed by improvements in survey design. Finally, data are aggregated to the national level for all estimates, and sub-national analyses may present a different picture. Further research is needed to examine within-country differences in birth registration by sociodemographic status and by region.

## Conclusions

This is the first paper to present disaggregated coverage estimates for birth certification in a standardized way for a large sample of countries using nationally representative data. The analyses are based on all children under five in the household, including children who are orphans. The use of multiple measures of inequality allows birth certificate coverage to be monitored for sub-groups, and allows governments, donors and policy makers to track progress on birth certificate coverage across urban/rural location, wealth groups, and sex of the child, which is necessary to improve access to birth registration, and to strengthen national data systems which can be used to monitor and address health inequalities.

Our results have several implications. First, efforts to improve CRVS systems must aim to reduce inequalities in birth certificate coverage within and between countries. There is an urgent need to focus on policies and interventions to address the fact that poor and rural children are denied their basic right to identity and are missing in national data. Without such efforts, CRVS systems will remain unable to fully contribute to policy and planning, and unable to ensure children under five can benefit from the services and opportunities tied to a birth certificate. Further research is needed to better understand the effects of weak birth registration on estimates of mortality, and to continue to document the inequities in these systems particularly as efforts to improve these systems result in average coverage increasing. As data needs for the Sustainable Development Goals expand, analyses which examine which children are missing in global health data are essential to improve birth certificate coverage and strengthen CRVS systems, in order to produce nationally representative health data, monitor inequality, and ensure accountability.

## Additional files

Additional file 1: Survey Questions on Birth Registration used to construct the primary and secondary outcomes. (DOCX 104 kb)

Additional file 2: Equity analyses and reference groups used in the analysis. (DOCX 53 kb)

Additional file 3: Birth Registration Status of Children Under Age Five in 94 countries: country level estimates, sample size, and data sources. (XLSX 27 kb)

Additional file 4: Slope Index of Inequality in Birth Certificate Coverage by Wealth Quintile Among Children Under Five. (DOCX 991 kb)

Additional file 5: Absolute Difference in Birth Certificate Coverage between Urban and Rural Residence Among Children Under Five. (DOCX 755 kb)

Additional file 6: Absolute Difference in Birth Certificate Coverage between Boys and Girls Under Five. (DOCX 660 kb)

## Abbreviations

CEE & CIS: Central and Eastern Europe and the Commonwealth of Independent States
CRVS: Civil Registration and Vital Statistics
DHS: Demographic health survey
LAC: Latin America and the Caribbean
MICS: Multi-indicator cluster survey
SDGs: Sustainable development goals
SII: Slope index of inequality
UNICEF: United Nations Children’s Fund

## References

[1] Dunning C, Gelb A, Raghavan S. Birth Registration, Legal Identity, and the Post-2015 Agenda. Washington, DC: Center for Global Development; 2014.

[2] Plan International (2009). Count every child: the right to birth registration.

[3] World Bank and WHO, Global Civil Registration and Vital Statistics. Scaling up investment plan 2015-2024. p. 2014.

[4] United Nations (1990). The convention on the rights of the child.

[5] United Nations News Service. UN News - UN lauds Somalia as country ratifies landmark children’s rights treaty. 2015. http://www.un.org/apps/news/story.asp?NewsID=49845#.WT7bhBPyuRs. Accessed 12 Jun 2017.

[6] UNICEF. A Passport To Protection: A Guide to Birth Registration Programming. New York: UNICEF; 2013.

[7] Ahmad OB, Lopez a D, Inoue M (2000). The decline in child mortality: a reappraisal. Bull World Health Organ.

[8] AbouZahr C, de Savigny D, Mikkelsen L, Setel PW, Lozano R, Nichols E (2015). Civil registration and vital statistics: progress in the data revolution for counting and accountability. Lancet.

[9] Gelb A, Dahan M. Means versus Ends: Deconstructing the SDGs and the Role of Identification | Center For Global Development. Center for Global Development. 2015. http://www.cgdev.org/blog/means-versus-ends-deconstructing-sdgs-and-role-identification. Accessed 8 Mar 2015.

[10] General Assembly. Transforming Our World: The 2030 Agenda for Sustainable Development A/RES/70/1. United Nations; 2015.

[11] UNICEF (2013). Every Child’s birth right: inequities and trends in birth registration.

[12] The DHS Program - Quality information to plan, monitor and improve population, health, and nutrition programs. http://dhsprogram.com/.

[13] Home - UNICEF MICS. http://mics.unicef.org/.

[14] Information by country | UNICEF. https://www.unicef.org/infobycountry/.

[15] World Bank Country and Lending Groups – World Bank Data Help Desk. https://datahelpdesk.worldbank.org/knowledgebase/articles/906519-world-bank-country-and-lending-groups.

[16] Barros AJD, Victora CG. Measuring coverage in MNCH: determining and interpreting inequalities in coverage of maternal, newborn, and child health interventions. PLoS Med. 2013;10. 10.1371/journal.pmed.1001390 PMC3646214 23667332

[17] Shea OR, Johnson K. The DHS wealth index: comparative reports No. 6. Calverton: ORC Macro; 2004.

[18] Filmer D, Pritchett LH (2001). Estimating wealth effects without expenditure data — or tears. Demography.

[19] World Health Organization. Handbook on health inequality monitoring: with a special focus on low-and middle-income countries. Geneva: World Health Organization; 2013.

[20] Jayaraman J, Roberts GJ, Wong HM, McDonald F, King NM. Ages of legal importance: implications in relation to birth registration and age assessment practices. Med Sci Law. 2015;0:1–6. 10.1177/0025802415590172 26101440

[21] AbouZahr C, de Savigny D, Mikkelsen L, Setel PW, Lozano R, Lopez AD. Towards universal civil registration and vital statistics systems: The time is now. Lancet. 2015;386:1407–18. 10.1016/S0140-6736(15)60170-2 25971217

[22] Harbitz M, Boekle-Giuffrida B. Democratic Governance, Citizenship, and Legal Identity: Linking Theoretical Discussion and Operational Reality. Washington, DC: Inter American Development Bank; 2009.

[23] Cappa C, Gregson K, Wardlaw T, Bissell S (2014). Birth registration: a child’s passport to protection. Lancet Glob Health.

[24] Phillips DE, AbouZahr C, Lopez AD, Mikkelsen L, de Savigny D, Lozano R (2015). Are well functioning civil registration and vital statistics systems associated with better health outcomes?. Lancet.

[25] Fagernäs S, Odame J. Birth registration and access to health care: an assessment of Ghana’s campaign success. Bull World Health Organ. 2013;91:459–64. 10.2471/BLT.12.111351 PMC3777139 24052683

[26] Brosco JP (1999). The early history of the infant mortality rate in America: “a reflection upon the past and a prophecy of the future”. Pediatrics.

[27] Mikkelsen L, Phillips DE, Abouzahr C, Setel PW, Savigny D De, Lozano R, et al. A global assessment of civil registration and vital statistics systems: monitoring data quality and progress. Lancet. 2015;6736:1395–406. 10.1016/S0140-6736(15)60171-4 25971218

[28] Adi AE, Abdu T, Khan A, Rashid MH, Ebri UE, Cockcroft A (2015). Understanding whose births get registered: a cross sectional study in Bauchi and Cross River states, Nigeria. BMC Res Notes.

[29] Amo-Adjei J, Annim SK (2015). Socioeconomic determinants of birth registration in Ghana. BMC Int Health Hum Rights.

[30] UNICEF. Ethiopia: Vital events registration launched – UNICEF Ethiopia. 2016. https://unicefethiopia.org/2016/08/25/ethiopia-conventional-vital-events-registration-launched/. Accessed 29 Jun 2017.

[31] National Institute of Statistics of Rwanda, National Identification Agency, Ministry of Local Government, Ministry of Health, Ministry of Justice, Ministry of Gender and Family Promotion (2016). Rwanda civil registration and vital statistics systems: comprehensive assessment final report.

[32] Landinfo. Documents in Somalia and Sudan. Oslo; 2009.

[33] Apland K, Hamilton C, Blitz BK, Lagaay M, Lakshman R, Yarrow E. Birth registration and children's rights: A complex story. Woking: Plan International; 2014.

[34] Victora CG, Vaughan JP, Barros FC (2000). Silva a C, Tomasi E. Explaining trends in inequities: evidence from Brazilian child health studies. Lancet.

[35] World Health Organization. The world health report 2003: Shaping the future. Geneva: World Health Organization; 2003.

[36] Mariana Muzzi. Good Practices in Integrating Birth Registration into Health Systems (2000–2009); Case Studies: Bangladesh, Brazil, the Gambia and Delhi, India. New York: UNICEF; 2010.

[37] Krieger N (2008). Who and what is a “population”? Historical debates, current controversies, and implications for understanding “population health” and rectifying health inequities. Milbank Q.

[38] Boerma JT, Stansfield SK (2007). Health statistics now: are we making the right investments?. Lancet.

