# Supplementary material for: Who and where are the uncounted children? Inequalities in birth certificate coverage among children under five years in 94 countries using nationally representative household surveys
Source: Int J Equity Health. 2017 Aug 18;16:148. doi: 10.1186/s12939-017-0635-6 (PMC5562988; doi:10.1186/s12939-017-0635-6)
Supplement: Supplementary file 1 — Survey Questions on Birth Registration used to construct the primary and secondary outcomes. (DOCX 104 kb) [file 12939_2017_635_MOESM1_ESM.docx]

**Additional File 1: Survey Questions on Birth Registration**

| **Demographic Health Survey Questions in DHS 5 (2003-2008) and DHS 6 (2008-2013):** |
| --- |
| “If age 0-4 years, does (NAME) have a birth certification? If NO, PROBE: Has (NAME)’s birth ever been registered with the civil authority?  1=Has certificate  2=Registered  3=Neither  8=Don’t know |

| **Multiple Indicator Cluster Survey Questions in MICS 4 and 5** | | |
| --- | --- | --- |
| **Birth registration BR** | | |
| **BR1**. Does *(name)* have a birth certificate?  *If yes, ask:*  May I see it? | Yes, seen 1  Yes, not seen 2  No 3  DK 8 | 1⇨Next  Module  2⇨Next  Module |
| **BR2**. Has (*name*)’s birth been registered with ***the civil authorities***? | Yes 1  No 2  DK 8 | 1⇨Next  Module |
| **BR3**. Do you know how to register (*name*)’s birth? | Yes 1  No 2 |  |

| **Multiple Indicator Cluster Survey Questions in MICS 3** | | |
| --- | --- | --- |
| birth registration and early learning module BR | | |
| BR1. Does (name) have a birth certificate? May I see it? | Yes, seen 1  Yes, not seen 2  No 3  DK 8 | 1⇨BR5 |
| BR2. Has (name’s) birth been registered with the civil authorities? | Yes 1  No 2  DK 8 | 1⇨BR5  8⇨BR4 |
| BR3. Why is (name’s) birth not registered? | Costs too much 1  Must travel too far 2  Did not know it should be registered 3  Did not want to pay fine 4  Does not know where to register 5  Other (specify) 6  DK 8 |  |
| BR4. Do you know how to register your child’s birth? | Yes 1  No 2 |  |

Notes:

- *MICS 3 uses a different skip pattern. If the response to BR1 is “Yes, not seen” BR2 is asked. In MICS 4 and 5, if the response to BR1 is “Yes, not seen”, the module is considered complete. The skip pattern from MICS 4 and 5 was applied to MICS 3.*
- *BR3 and BR4 were not used in the analysis*
