# Supplementary material for: Who and where are the uncounted children? Inequalities in birth certificate coverage among children under five years in 94 countries using nationally representative household surveys
Source: Int J Equity Health. 2017 Aug 18;16:148. doi: 10.1186/s12939-017-0635-6 (PMC5562988; doi:10.1186/s12939-017-0635-6)
Supplement: Supplementary file 2 — Equity analyses and reference groups used in the analysis. (DOCX 53 kb) [file 12939_2017_635_MOESM2_ESM.docx]

**Additional File 2: Equity analyses and reference groups**

| *Stratifier* | *Categories* | *Reference group* | *Measure of inequality* | *Interpretation* |
| --- | --- | --- | --- | --- |
|  |  |  |  |  |
| Wealth Quintile | Poorest  (wealth quintile 1) to  Richest  (wealth quintile 5) | Richest (wealth quintile 5) | Slope Index of Inequality | Positive values indicate a higher birth certificate coverage among children in the richest wealth quintile compared to the poorest. A small SII shows small differences between the poorest wealth quintile compared to the richest. |
| Sex | Male, Female | Male | Absolute difference  *(Female-Male)* | Negative values indicate lower birth certificate coverage among girls compared to boys |
| Residence | Urban, Rural | Urban | Absolute difference  *(Rural-Urban)* | Negative values indicate lower birth certificate coverage among children in rural areas compared to urban areas |
