# Supplementary material for: Who and where are the uncounted children? Inequalities in birth certificate coverage among children under five years in 94 countries using nationally representative household surveys
Source: Int J Equity Health. 2017 Aug 18;16:148. doi: 10.1186/s12939-017-0635-6 (PMC5562988; doi:10.1186/s12939-017-0635-6)
Supplement: Supplementary file 4 — Slope Index of Inequality in Birth Certificate Coverage by Wealth Quintile Among Children Under Five. (DOCX 991 kb) [file 12939_2017_635_MOESM4_ESM.docx]

**Additional File 4: Slope Index of Inequality in Birth Certificate Coverage by Wealth Quintile Among Children Under Five**

Notes: *Figure shows point estimates and confidence intervals, and zero indicates the null value. Positive values show a “pro-rich” bias – a higher birth certificate coverage among children in the richest wealth quintile compared to the poorest.*


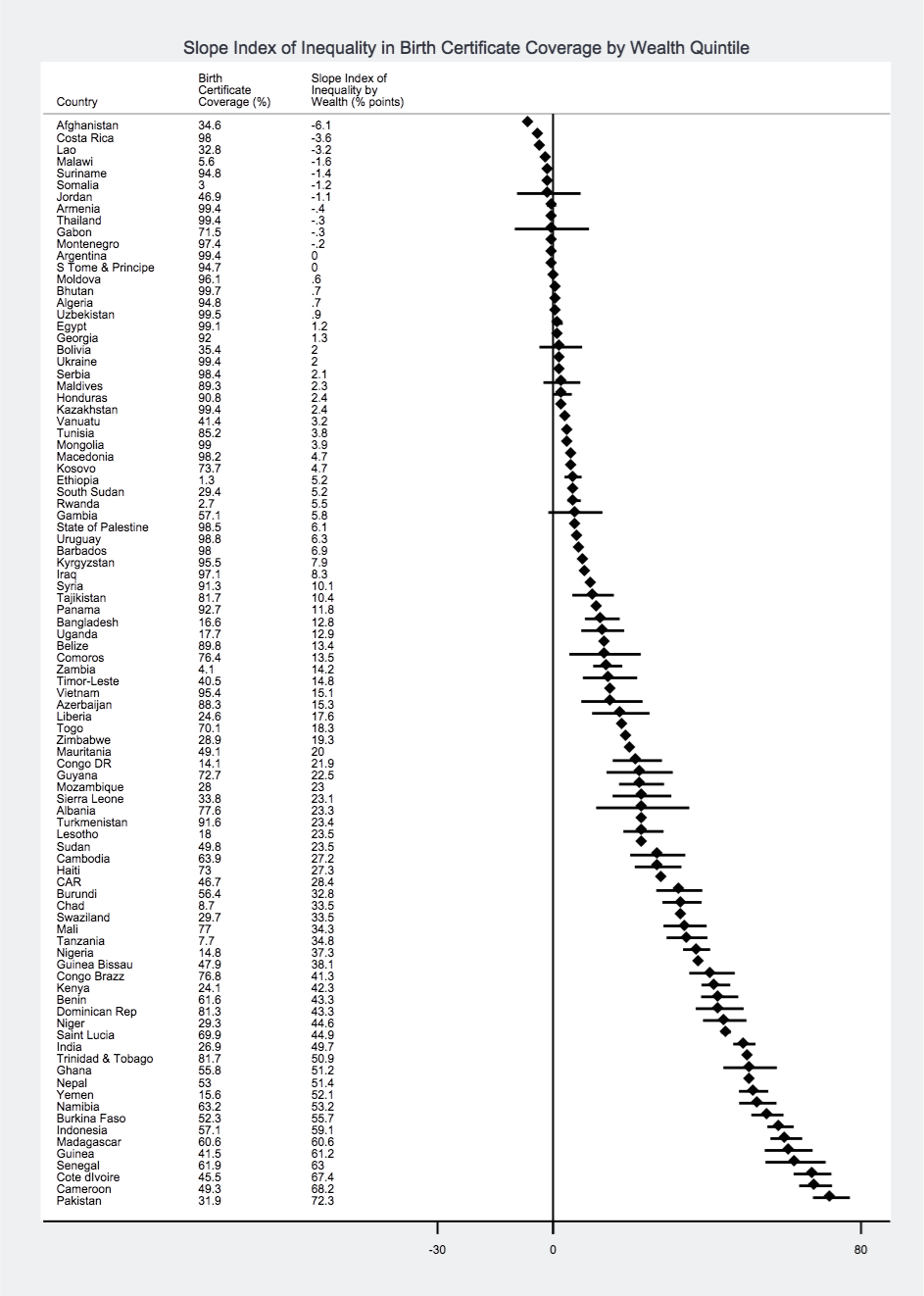


SII (% points)
