# Supplementary material for: Who and where are the uncounted children? Inequalities in birth certificate coverage among children under five years in 94 countries using nationally representative household surveys
Source: Int J Equity Health. 2017 Aug 18;16:148. doi: 10.1186/s12939-017-0635-6 (PMC5562988; doi:10.1186/s12939-017-0635-6)
Supplement: Supplementary file 6 — Absolute Difference in Birth Certificate Coverage between Boys and Girls Under Five. (DOCX 660 kb) [file 12939_2017_635_MOESM6_ESM.docx]

**Additional File 6: Absolute Difference in Birth Certificate Coverage between Boys and Girls Under Five**

Notes: *Figure shows point estimates and confidence intervals, and zero indicates the null value. Negative values indicate a “pro-male” bias – a lower birth certificate coverage among girls compared to boys*


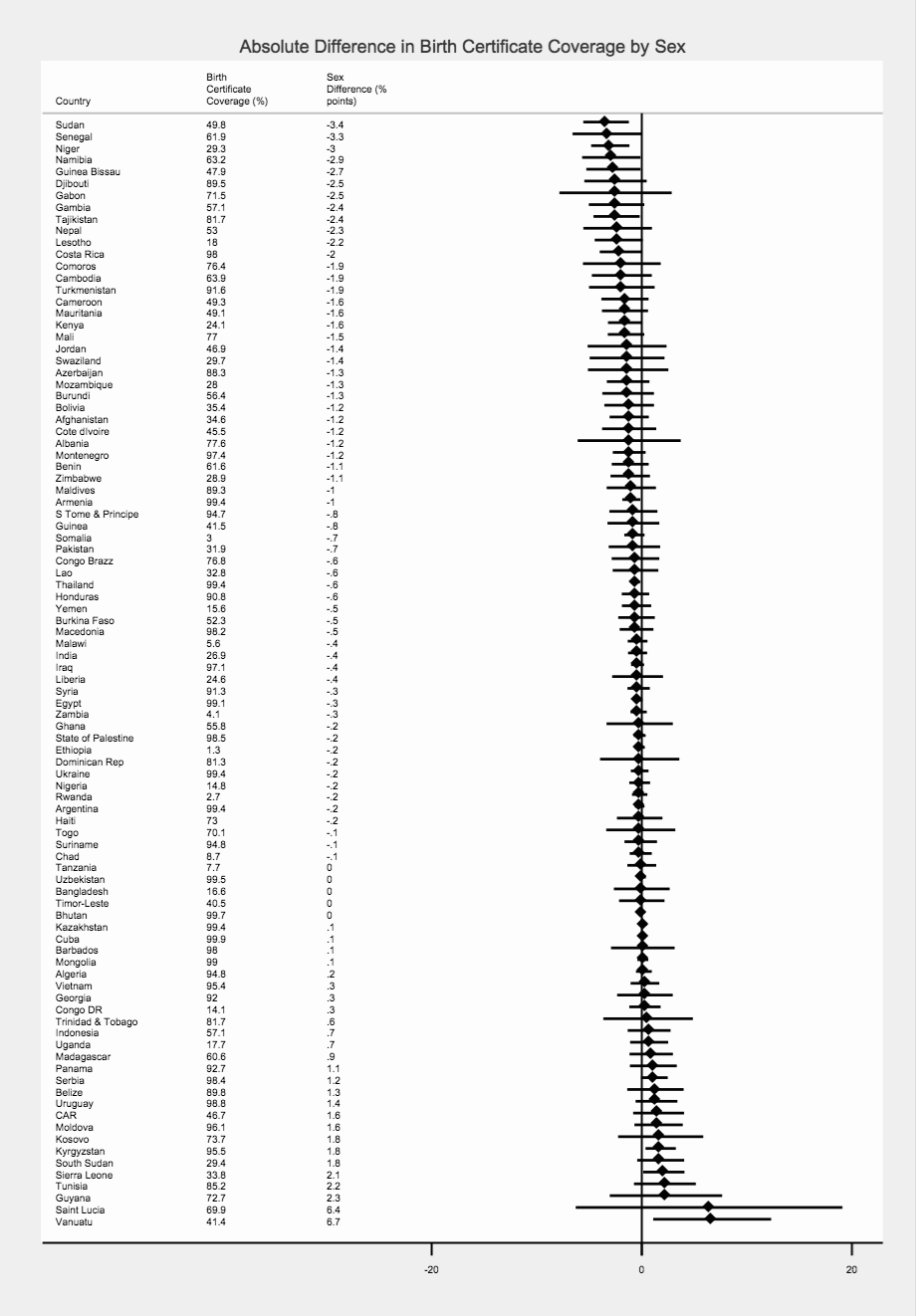


Absolute Difference (% points)
